# Manifestation of connective tissue diseases (CTD) in pediatrics patients with autoimmune hepatitis (AIH)

**DOI:** 10.1186/1546-0096-9-S1-P232

**Published:** 2011-09-14

**Authors:** S Garay, M Mastri, A Besga, M Fabi, T Gonzalez Villar

**Affiliations:** 1 Rheumatology and Gastroenterology Units, Hospital “Sor M. Ludovica”, La Plata, Buenos Aires, Argentina

## Background

Autoimmune hepatitis (AIH) is a progressive inflammatory fribrosing disease of the liver of unknown etiology, which in its normal progress leads to cirrhosis. It is characterized by the increase in aminotranferases and hypergammaglobulinemia, and the presence of non-organ and specific liver autoantibodies in serum, which allow for its classification. Two subtypes are differentiated: Type I AIH with anti-smooth muscle antibodies (SMA) and/or antinuclear antibodies (ANA); and Type II AIH with antimichondrial antibodies (LKM).

AIH can be associated with other autoimmune illnesses such as thyroiditis, Type I diabetes, thrombocytopenia, hemolytic anaemia and ulcerative colitis. Likewise, the association to connective tissue diseases (CTD) has been communicated, such as Systemic Lupus Erythematosus, Sjögren’s Syndrome, Undifferentiated Connective Tissue Disease, Mixed Connective Tissue Disease, Localized Sclerodermia and Juvenile Idiopathic Arthritis.

## Aim

We aim to research defined or probable clinical and laboratory manifestations of CTD in patients with AIH diagnosis in pediatric age.

## Methods

20 records of patients with AIH diagnosis under care in the Hepatology Unit and Rheumatology Unit were analyzed retrospectively since 1995 until May 2008.

## Results

In the group of 20 patients, 6 were male (30%) and 14 were female (70%). Their average AIH diagnosis age was 9.6 yrs (Min 1.4 yrs-Max 15.2 yrs). Type I AIH diagnosis occurred in 18/20 patients (90%), 5 males (27.8%) and 13 females (72.2%), their average diagnosis age being 9.8 yrs (Min 1.4 yrs-Max 15.2 yrs), and Type II AIH diagnosis occurred in 2/20 patients (10%), 1 male and 1 female. Their average AIH diagnosis age was 7.5 yrs (Min 4.2 yrs-Max 10.9 yrs). Average follow-up period of the patients until the end of the study was 41.4 months in average.

8/20 patients with defined AIH (40%) showed some manifestations of CTD in their progress; 6/18 patients with Type I AIH (33.3%), 2 males and 6 females, and 2/2 patients with Type II AIH (100%). Most common manifestations were: arthralgia/arthritis, vasculitis, AHA with positive Coombs’ Test results, oral ulcers, hypocomplementemia, leukopenia/lymphopenia and positive ANA results. CTD defined diagnosis were: SLE, vasculitis, Sjörgren’s Syndrome and Overlap Syndrome. In the remaining 4 patients diagnosis was probable. In one patient manifestations of CTD were simultaneous with AIH diagnosis. In 3/8 patients manifestations were before (25 months average) and in 4/8 were afterwards (29 months average).

## Conclusions

In 8 out of 20 patients AIH was associated with defined or probable CTD. Three of the 8 patients developed AIH after CTD diagnosis.

This association should be investigated in all patients with AIH and CTD diagnosis.

